# The Latest Overview of circRNA in the Progression, Diagnosis, Prognosis, Treatment, and Drug Resistance of Hepatocellular Carcinoma

**DOI:** 10.3389/fonc.2020.608257

**Published:** 2021-02-17

**Authors:** Dandan Xiong, Rongquan He, Yiwu Dang, Huayu Wu, Zhenbo Feng, Gang Chen

**Affiliations:** ^1^ Department of Pathology, First Affiliated Hospital of Guangxi Medical University, Nanning, China; ^2^ Department of Medical Oncology, First Affiliated Hospital of Guangxi Medical University, Nanning, China; ^3^ Department of Cell Biology & Genetics, School of Preclinical Medicine, Guangxi Medical University, Nanning, China

**Keywords:** hepatocellular carcinoma, circular RNAs, biological function, biomarker, treatment, drug resistance

## Abstract

Hepatocellular carcinoma (HCC) is one of the main causes of tumor-related deaths worldwide. Due to the lack of obvious early symptoms and the lack of sensitive screening indicators in the early stage of HCC, the vast majority of patients are diagnosed with advanced or metastatic HCC, resulting in dissatisfactory treatment result. Therefore, it is urgent to determine effective and sensitive diagnostic and prognostic indicators and to determine new therapeutic targets. Circular RNA (circRNA) is a type of non-coding RNA that has been neglected for a long time. In recent years, it has been proved to play an important role in the development of many human diseases. Increasing evidence shows that change in circRNA expression has an extensive effect on the biological behavior of HCC. In this study, we comprehensively tracked the latest progress of circRNA in the pathogenesis of HCC, and reviewed its role as a biomarker for diagnosis and prognosis prediction in patients with HCC. In addition, we also summarized the potential of circRNA as therapeutic target in HCC and its relationship with HCC drug resistance, providing clues for the clinical development of circRNA-based therapeutic strategies.

## Introduction

Hepatocellular carcinoma (HCC) is the fourth leading cause of tumor-related deaths worldwide, causing approximately 78,200 deaths per year. In addition, it is estimated that there are 841,000 new cases of HCC worldwide each year, ranking seventh among all malignancies (1). Although treatment is improved in recent years, patients with HCC suffer unfavorable prognosis. The main reason is that the symptoms in early HCC are not obvious and there is a lack of practical diagnostic markers, leading to the great majority of patients are diagnosed with advanced or metastatic HCC. For these patients, the efficacy of local radiotherapy or chemotherapy is unsatisfactory, and there is little chance of curing by surgery. Thus, it is urgent to identify effective and sensitive diagnostic and prognostic markers, and to develop new treatment strategies for patients with HCC.

In recent years, a novel class of RNA molecule, circular RNA (circRNA), has received increasing attention due to its essential role in various diseases (2, 3), including human malignant tumors (4–7). CircRNA is formed by covalent closure of the 5′ and 3′ ends of the precursor messenger RNA (pre-mRNA) through back splicing (8). It contains a closed-loop structure that makes it resistant to digestion and cleavage of exonucleases, thus being highly stable in tissues and cells (9, 10). Additionally, most circRNAs exhibit high tissue- and cell-specific expression patterns and high conservation among species (11, 12). On the basis of these characteristics, circRNAs present great potential as indicators for disease diagnosis, progress monitoring and prognosis prediction in the clinic (13, 14).

Relying on the advancement of high-throughput sequencing and circRNA-related computational technologies, the biological function and clinical value of circRNA are gradually being explored. Emerging evidence suggests that circRNA involves in tumorigenesis. Li et al. (15) report that circACC1 promotes tumor cell growth by activating the AMPK complex under metabolic stress. Chen et al. (16) demonstrate that circRNA FECR1 promotes tumor deterioration by regulating DNA methylation and demethylating enzymes. Su et al. (17) indicate that circRNA cTFRC functions as a sponge of miR-107 to facilitate bladder cancer development. The critical role of circRNA in tumorigenesis makes it a prospective therapeutic target for cancer patients. Here, we summarized latest research on circRNA in HCC to outline the biological function, mechanism of action and clinical value of circRNA in HCC. The final goal of this review is to yielded more effective strategies for the diagnosis and prognosis prediction of HCC and to provide a basis for circRNA-based HCC molecular targeted therapy.

## Biogenesis of circRNA

**Figure 1:**
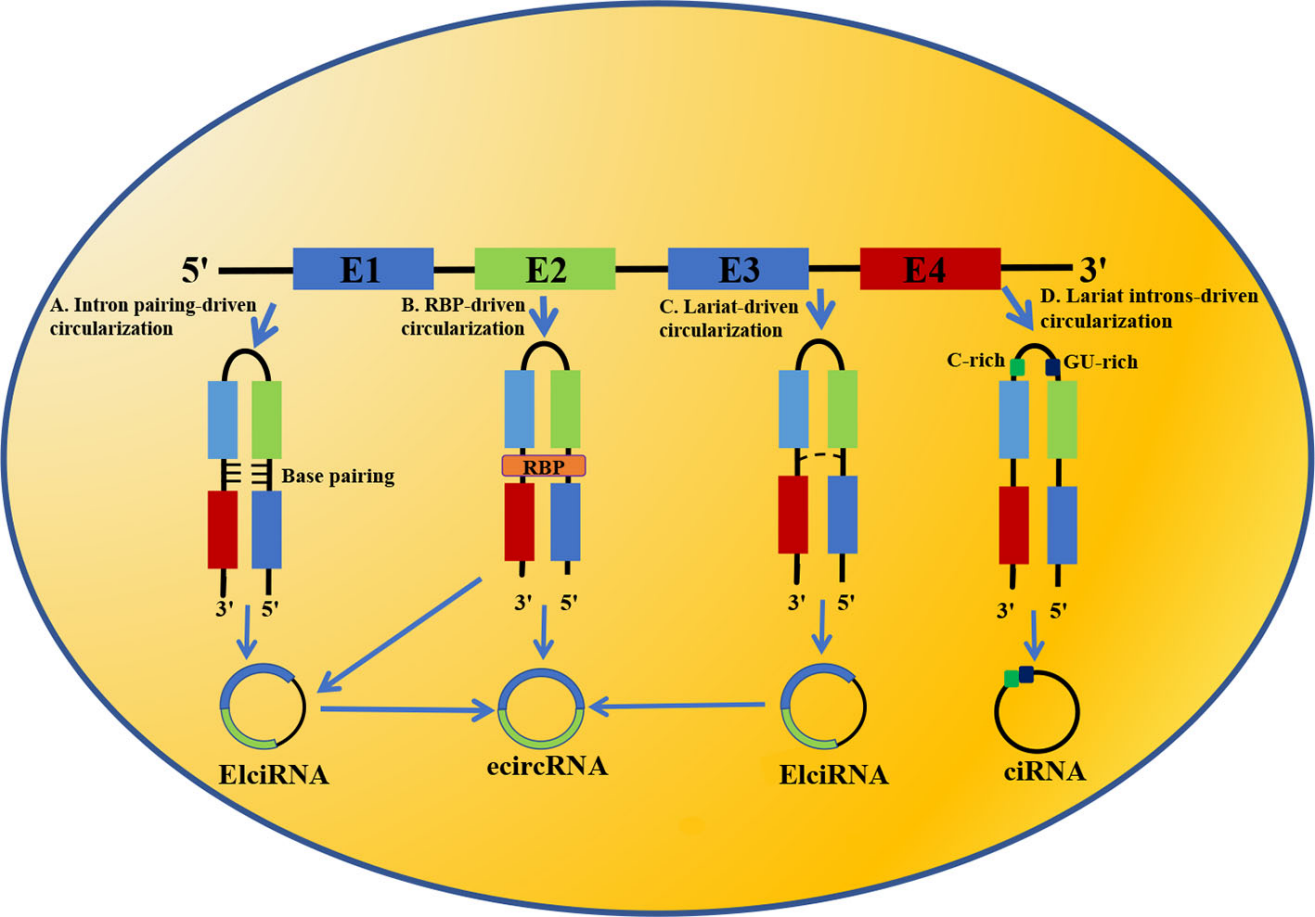
Biogenesis of circRNA. **(A)** Intron pairing-driven circularization; **(B)** RNA binding protein (RBP)-driven circularization; **(C)** Lariat-driven circularization; **(D)** Lariat introns-driven circularization.

**Figure 2:**
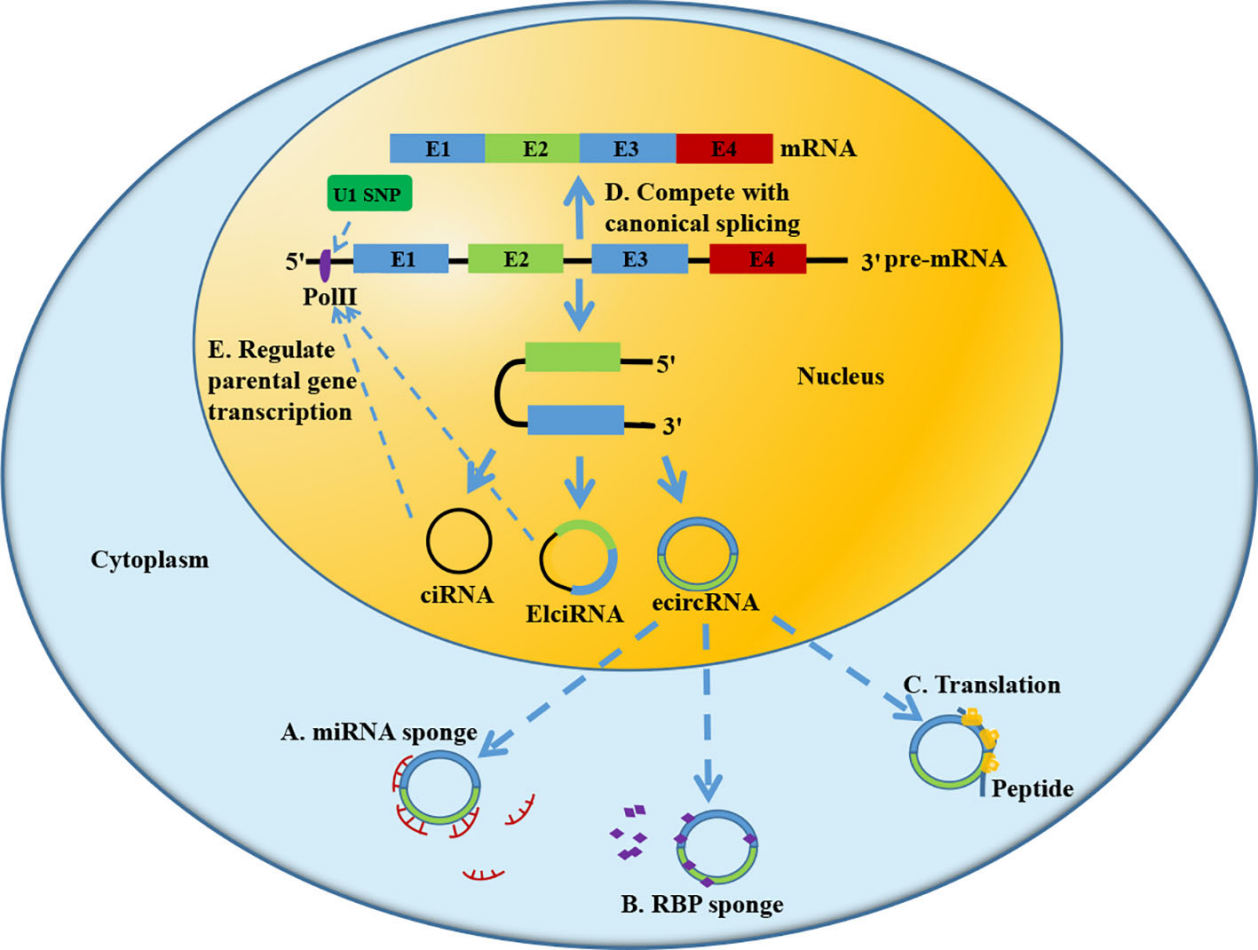
Biological function of circRNA. **(A)** Acting as miRNA sponge; **(B)** Combining with RNA binding protein (RBP); **(C)** Translating protein; **(D)** Competing with splicing of pre-mRNA. **(E)** Regulating transcription of parental gene.

**Figure 3:**
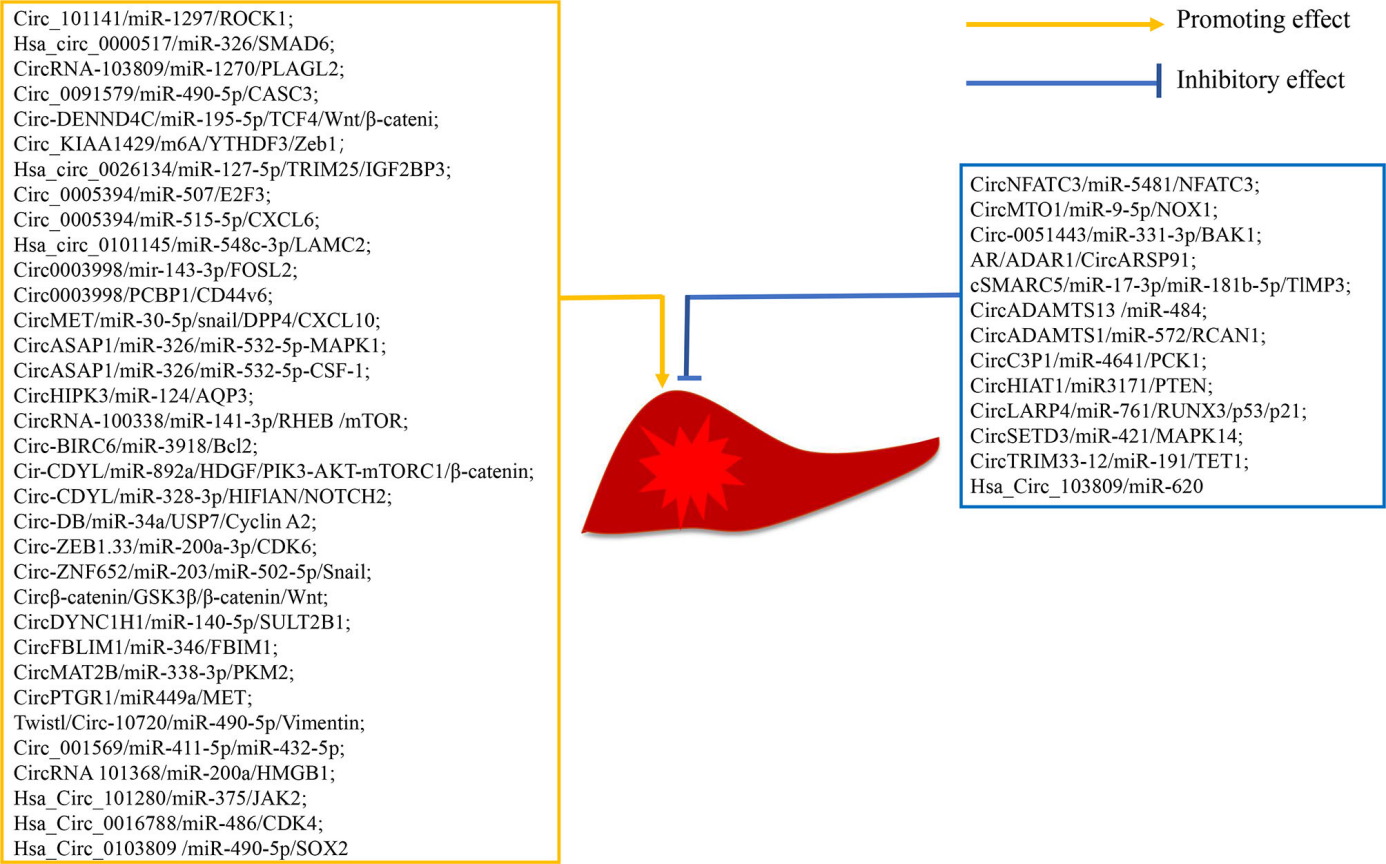
Mechanism of action of circRNAs in hepatocellular carcinoma.

**Figure 4:**
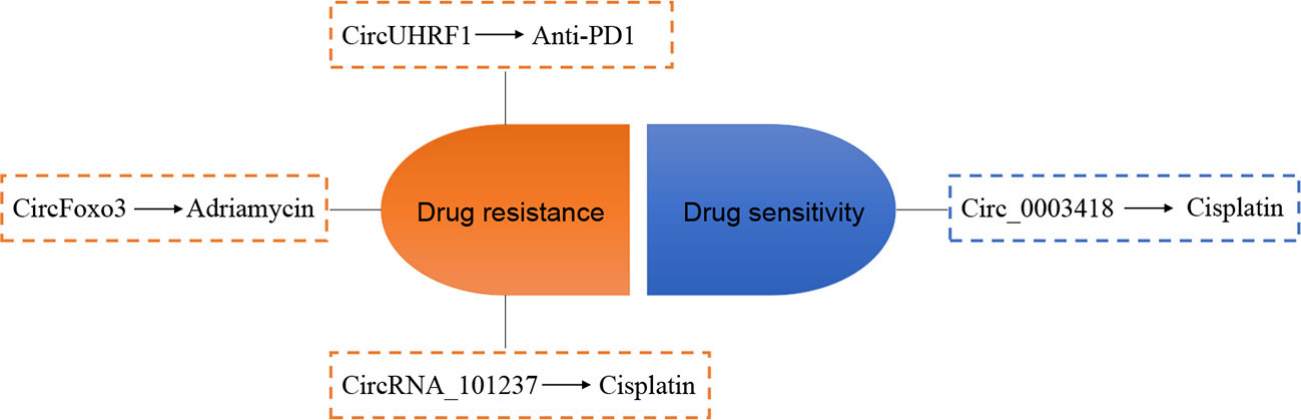
Relationship between circRNA and chemotherapy resistance in hepatocellular carcinoma (HCC). The arrows indicate promotion effect.

Up to now, the formation mechanism of circRNA has not been clearly understood. Majority of scientists consider that the biogenesis of circRNA differs from the linear mRNA: linear mRNA is produced by processing exons of pre-mRNA by canonical splice, while circRNA is formed by back-splicing and is regulated by cis-acting and trans-acting elements. Nowadays, the circularization mechanisms of circRNA have been suggested: (1) Intron pairing-driven circularization (**Figure 1A**); (2) RNA-binding protein (RBP)-driven circularization (**Figure 1B**); (3) Lariat-driven circularization (**Figure 1C**); (4) Lariat introns-driven circularization (**Figure 1D**).

### Intron Pairing-Driven Circularization

In this circularization model, the flanking introns of circRNA harbor reverse complementary sequences, resulting in base pairing, which shorten the distance between introns and connect the downstream 5′ splice site to the upstream 3′ splice site of pre-mRNA, thereby forming exonic circRNA (ecircRNA) or exonic-intronic circRNA (EIciRNA). Intron pairing-driven circularization depends on 30~40nt reverse complementary repeat elements, including reverse complementary matches (RCMs), intron complementary sequences (ICMs) and Alu elements. The foremost of these is Alu element, which is the main factor that facilitates intron pairing and mediates circularization in the mammalian genome, especially in the human genome (18, 19). Meanwhile, the competitive pairing between reserve complementary repeat elements leads to alternative splicing and thus promotes the diversity of circRNA.

Notably, not all introns can facilitate the back-splicing of pre-mRNA and looping of exons. For instance, base-pairing may cause hairpin structure, resulting in failure to form circRNA (20).

### RBP-Driven Circularization

By binding to the specific motif of an exon-flanking intron or forming dimers, RBP brings flanking introns together to regulate the looping of exons, thereby facilitating the formation of ecircRNA or EIciRNA. For instance, a previous study demonstrates that quaking (QKI), an RBP, regulate circRNA formation by binding to flanking introns and subsequently bringing downstream and upstream of the circRNA-forming exons together (21).

### Lariat-Driven Circularization

In the lariat-driven circularization mechanism, the upstream 3′ splicing receptor of pre-mRNA covalently attaches to the downstream 5′ splicing donor to form a lariat intermediate, which is composed of several exons and introns. Provided the introns are not completely spliced out but retained in the lariat, the lariat is likely to transform into ElciRNA. If all introns are removed, exons within lariat will form a connection by 3′, 5′-phosphodiester bond, thereby generating ecircRNA.

### Lariat Introns-Driven Circularization

After the process of pre-mRNA splicing, a majority of introns are degraded. However, the minority of introns which have a 7nt-GU-rich motif in the 5′ splicing site and an 11nt-C-rich motif in the 3′ branchpoint site may escape degradation by debranching enzyme, resulting in the generation of circular intronic RNA (ciRNA).

## Biological Function of circRNA

Based on different formation mechanism and different components, circRNA is mainly divided into three subtypes: ecircRNA, EIciRNA, and ciRNA. EcircRNA consists of single or several exons and is primarily located in the cytoplasm. Previous evidence demonstrates that ecircRNA mainly functions in human tissues by acting as miRNA sponge (22) (**Figure 2A**), interacting with RBP (23) (**Figure 2B**) and translating protein (24) (**Figure 2C**). EIciRNA and ciRNA are intron-containing circRNAs, which are mainly located in the nucleus. They have the potential to compete with linear mRNA splicing (25) (**Figure 2D**) and to regulate the transcription of parental genes (26, 27) (**Figure 2E**). Nowadays, most studies focus on the regulatory role of circRNA as a miRNA sponge in HCC. Further in-depth studies are needed to explore other mechanism by which circRNA affects HCC development.

## Role of circRNA in HCC

### CircRNA Promotes Tumor Progression in HCC

In the long-term study of circRNA in HCC, researchers have found that some circRNAs promote the progression of HCC (**Table 1** and **Figure 3**). Zhang et al. (28) demonstrate that circ_101141 accelerates the progression of HCC by sponging miR-1297 and regulating ROCK1 expression. He et al. (29) show that hsa_circ_0000517 is upregulated in HCC, and silencing hsa_circ_0000517 arrests HCC development by mediating the miR-326/SMAD6 axis. Cao et al. (30) find that silencing circRNA-103809 in HCC cells inhibits cell proliferation, migration, invasion, and epithelial-to-mesenchymal transition (EMT). Circ_0091579 accelerates HCC cell proliferation, migration, invasion, and glycolysis by targeting the miR-490-5p/CASC3 axis (31). Circ-DENND4C, an onco-circRNA in breast cancer, is overexpression in HCC and increase the expression of TCF4 to activate the Wnt/β-catenin signaling cascade by sponging miR-195-5p (32). Hsa_circ_0084922, also known as circ_KIAA1429, is upregulated in HCC, and facilitates HCC advancement by upregulating Zeb1 in a m6A-YTHDF3 dependent manner (33). Hsa_circ_0026134 is overexpressed in HCC and promotes HCC growth and metastasis by sponging miR-127-5p to upregulate TRIM25 and IGF2BP3 (34). Sun et al. (35) demonstrate that circ_0005394 facilitates HCC growth *via* mediating the miR-507/E2F3 and miR-515-5p/CXCL6 axes. Jin et al. (36) find that hsa_circ_0101145 is overexpressed in HCC and silencing hsa_circ_0101145 inhibits the deterioration of HCC by targeting the miR-548c-3p/LAMC2 axis. Circ0003998, upregulated in portal vein tumor thrombus HCC tissues, is identified to promote HCC EMT by acting as miR-143-3p sponge to promote an EMT-related stimulator, FOSL2. Additionally, circ0003998 can bind with PCBP1 to enhance the expression of an EMT-related gene, CD44v6 (37). A study conducted by Huang et al. (38) shows that circMET is an onco-circRNA that promotes HCC evolvement and induces immune suppression by the Snail/DPP4/CXCL10 axis. Hu et al. (39) carry out a circRNA sequencing analysis on 15 samples from HCC patients with postoperative lung metastasis and 15 without metastasis or recurrence and determine circASAP1 as a metastasis-related circRNA in HCC. Mechanism analysis shows that circASAP1 accelerate HCC metastasis by targeting the miR-326/miR-532-5p-MAPK1 axis. Additionally, circASAP1 regulates tumor-associated-macrophage (TAM) infiltration by mediating the miR-326/miR-532-5p-CSF-1 axis. CircHIPK3 is overexpressed in HCC tissues, and knockdown of circHIPK3 decreases cell proliferation and migration by targeting miR-124 to regulate the expression of AQP3 (40). Huang et al. (41) find that circRNA-100338 is overexpressed in HCC tissues and enhances HCC proliferation and migration by modulating the miR-141-3p/RHEB axis to activate mTOR signaling pathway. Also, circRNA-100338 is upregulated in both highly metastatic HCC cells and their secreted exosomes, and overexpressed or silencing exosomal circRNA-100338 remarkably promotes or reduces HCC invasion, indicating that circRNA-100338 is a metastasis-related circRNA in HCC (42). Circ-BIRC6 is upregulated in HCC tissues and HCC cells. Overexpression of circ-BIRC6 enhances HCC cell proliferation, migration and invasion, and inhibits HCC cell apoptosis by targeting the miR-3918/Bcl2 axis (43). A study conducted by Wei et al. (44) shows that circ-CDYL is upregulated in early HCC and contributes to the chemoresistance and tumorigenic properties of HCC. Therapies combining circ-CDYL interference and traditional enzyme inhibitors targeting phosphoinositide 3-kinase (PI3K) and hypoxia-inducible factor asparagine hydroxylase (HIF1AN) effectively inhibit tumor growth in HCC. Zhang et al. (45) report that the exosome circ-DB secreted by adipocytes promotes HCC cell proliferation and reduces DNA damage by reducing miR-34a and inducing the USP7/Cyclin A2 signaling cascade. Gong et al. (46) show that Circ-ZEB1.33 facilitates HCC cell proliferation by promoting cells into S phase. Guo et al. (47) find that circ-ZNF652 is upregulated in HCC. Depletion of circ-ZNF652 remarkably inhibits the migration and invasion of HCC cells *in vitro*, and suppresses HCC metastasis *in vivo* by binding to miR-203 and miR-502-5p to promote their common gene Snail, which is an important transcription factor that activates EMT. Liang et al. (48) reveal that circβ-catenin promotes HCC development by translation of a novel 370-amino acid β-catenin isoform that can stabilize β-catenin and subsequent activate of the Wnt pathway. CircDYNC1H1 has been reported to sponge miR-140-5p, and silencing circDYNC1H inhibits the proliferation and migration of HCC cells by down-regulating SULT2B1 (49). CircFBLIM1 is upregulated in HCC. Knockdown of circFBLIM1 suppresses the proliferation and invasion of HCC cells and promotes their apoptosis by modulating FBLIM1 *via* binding to miR-346  (50). Li et al. (51) demonstrate that circMAT2B facilitates glycolysis and tumor growth in HCC by regulating the miR-338-3p/PKM2 axis under hypoxia. Wang et al. (52) report that circPTGR1, which contains three isoforms of hsa_circ_0003731, hsa_circ_0008043 and has_circ_0088030, is upregulated in serum exosomes of HCC patients. Cell migration and invasion are significantly inhibited in HCC cells with exosomes from the circPTGR1-knockdown cells. The authors further clarify that circPTGR1 promotes HCC growth *via* the miR-449a-MET pathway. Meng et al. (53) demonstrate that circ-10720 is regulated by a transcription factor Twist. Overexpressed circ-10720 in HCC cells promotes cell proliferation, migration and invasion, and knockdown of circ-10720 in HCC cells inhibits cell migration and invasion. Liu et al. (54) report that knockdown of circ001569 significantly inhibits the growth, migration and invasion of Huh7 cells. Li et al. (55) find that circRNA101368 is overexpressed in HCC. Knockdown of circRNA101368 reduces HCC cell migration by regulating the miR-200a/HMGB1 axis. Study on hsa_circ_101280 shows that overexpressed hsa_circ_101280 promotes the proliferation and decreases the apoptosis of HCC cells *in vitro* by sponging miR-375 to upregulate JAK2 (56). Guan et al. (57) indicate that silencing hsa_circ_0016788 suppresses the proliferation, invasion and promotes the apoptosis of HCC cells *in vitro*, and inhibits tumor growth *in vivo*. Cai et al. (58) find that knockdown of hsa_circ_0103809 inhibits the proliferation and migration and induces the apoptosis of HCC cells by targeting the miR-490-5p/SOX2 axis. Our research group has been focusing on the role of circRNA in HCC. We have previously demonstrated that hsa_circ_0088364 and hsa_circ_0090049 are up-regulated in HCC cells (72). Hsa_circ_0088364 promotes HCC cells migration and inhibits HCC cells apoptosis. Has_circ_0090049 enhances cell proliferation and migration and suppresses cell apoptosis. Growing evidences indicate that some circRNAs, such as hsa_circ_00156 (73), hsa_circ_000224 (73), and hsa_circ_000520 (73), hsa_circ_0091579 (74), and hsa_circ_0128298 (75), are upregulated in HCC tissues or cells. But their biological functions or mechanisms of action still needed to be elucidated.

**Table 1:** Biological function and action mechanism of circRNA in hepatocellular carcinoma (HCC).

CircRNA	Expression change	Experiment	Cell line	Biological function	Regulatory axis	Ref.
Circ_101141	Upregulated in HCC	*In vitro and in vivo*	Hep3B, Huh7	Proliferation (+), invasion (+), migration (+)	Circ_101141/miR-1297/ROCK1	(28)
Hsa_circ_0000517	Upregulated in HCC	*In vitro* and *in vivo*	HCCLM3, Huh7	Proliferation (+), colony formation (+), migration (+), invasion (+)	Hsa_circ_0000517/miR-326/SMAD6	(29)
CircRNA-103809	Upregulated in HCC	*In vitro*	Huh7, HepG2	Proliferation (+), migration (+), invasion (+), EMT (+)	CircRNA-103809/miR-1270/PLAGL2	(30)
Circ_0091579	Upregulated in HCC	*In vitro*	HCCLM3, Huh7	Proliferation (+), invasion (+), migration (+), glycolysis (+)	Circ_0091579/miR-490-5p/CASC3	(31)
Circ-DENND4C	Upregulated in HCC	*In vitro* and *in vivo*	Huh7, HepG2	Proliferation (+), stemness (+), invasion (+), apoptosis (−)	Circ-DENND4C/miR-195-5p/TCF4/Wnt/β-catenin signaling pathway	(32)
Circ_KIAA1429	Upregulated in HCC	*In vitro* and *in vivo*	HepG2, Bel7404	Migration (+), invasion (+), EMT (+)	Circ_KIAA1429/m6A/YTHDF3/Zeb1	(33)
Hsa_circ_0026134	Upregulated in HCC	*In vitro* and *in vivo*	HepaRG, HepG2	Proliferation (+), invasion (+)	Hsa_circ_0026134/miR-127-5p/TRIM25/IGF2BP3	(34)
Circ_0005394	Upregulated in HCC	*In vitro*	Huh7, HepG2	Proliferation (+), migration (+), EMT (+)	Circ_0005394/miR-507/E2F3, circ_0005394/miR-515-5p/CXCL6	(35)
Hsa_circ_0101145	Upregulated in HCC	In vitro and *in vivo*	Huh7, HepG2	Proliferation (+), invasion (+), migration (+), apoptosis (−)	Hsa_circ_0101145/miR-548c-3p/LAMC2	(36)
Circ0003998	Upregulated in HCC	*In vitro* and *in vivo*	HepG2, MHCC97H	EMT (+)	Circ0003998/mir-143-3p/FOSL2, circ0003998/PCBP1/CD44v6	(37)
CircMET	Upregulated in HCC	*In vitro* and *in vivo*	HCCLM3, HepG2	Migration (+), invasion (+), EMT (+), immunosuppression (+)	CircMET/miR-30-5p/snail/DPP4/CXCL10	(38)
CircASAP1	Upregulated in metastatic HCC	*In vitro* and *in vivo*	HepG2, MHCC97H	Proliferation (+), colony formation (+), migration (+), invasion (+), TAM infiltration (+)	CircASAP1/miR-326/miR-532-5p-MAPK1, circASAP1/miR-326/miR-532-5p-CSF-1	(39)
CircHIPK3	Upregulated in HCC	*In vitro* and *in vivo*	Huh7, MHCC97H	Proliferation (+), metastasis (+)	CircHIPK3/miR-124/AQP3	(40)
CircRNA-100338	Upregulated in HCC	*In vitro*	BEL7402, Hep3B	Proliferation (+), metastasis (+)	CircRNA-100338/miR-141-3p/RHEB/mTOR signaling pathway	(41)
CircRNA-100338	Upregulated in metastatic HCC	*In vitro* and *in vivo*	Hep3B, MHCC97H	Invasion (+)		(42)
Circ-BIRC6	Upregulated in HCC	*In vitro* and *in vivo*	HepG2, Bel7402	Proliferation (+), invasion (+), migration (+), apoptosis (−)	Circ-BIRC6/miR-3918/Bcl2	(43)
Circ-CDYL	Upregulated in HCC	*In vitro* and *in vivo*	HCCLM3, SMMC7721	Proliferation (+)	Cir-CDYL/miR-892a/HDGF/PIK3-AKT-mTORC1/β-catenin signaling pathway, circ-CDYL/miR-328-3p/HIFlAN/NOTCH2 signaling pathway	(44)
Circ-DB	Upregulated in HCC	*In vitro*	HepG2, Hepa1-6	Proliferation (+), migration (+)	Circ-DB/miR-34a/USP7/Cyclin A2	(45)
Circ-ZEB1.33	Upregulated in HCC	*In vitro*	Huh7	Proliferation (+)	Circ-ZEB1.33/miR-200a-3p/CDK6	(46)
Circ-ZNF652	Upregulated in HCC	*In vivo*	HCCLM3, SK-HEP-1	Migration (+), invasion (+), EMT (+)	Circ-ZNF652/miR-203/miR-502-5p/Snail	(47)
Circβ-catenin	Upregulated in HCC	*In vitro*	Huh7, PLC-REF-5	Proliferation (+), invasion (+)	Circβ-catenin/GSK3β/β-catenin/Wnt signaling pathway	(48)
CircDYNC1H1	Upregulated in HCC	*In vitro*	SMMC7721, BEL-7402	Proliferation (+), migration (+)	CircDYNC1H1/miR-140-5p/SULT2B1	(49)
CircFBLIM1	Upregulated in HCC	*In vitro* and *in vivo*	HepG2, BEL7402, MHCC97H	Proliferation (+), invasion (+), apoptosis (−)	CircFBLIM1/miR-346/FBIM1	(50)
CircMAT2B	Upregulated in HCC	*In vitro* and *in vivo*	Huh7, HepG2	Proliferation (+), migration (+), invasion (+)	CircMAT2B/miR-338-3p/PKM2	(51)
CircPTGR1	Upregulated in HCC	*In vitro* and *in vivo*	HepG2, MHCC97L	Migration (+), invasion (+)	CircPTGR1/miR449a/MET	(52)
Circ-10720	Upregulated in metastatic HCC	*In vitro* and *in vivo*	PLC-REF-5, SMMC7721	Proliferation (+), migration (+), invasion (+), EMT (+)	Twistl/Circ-10720/miR-490-5p/Vimentin	(53)
Circ001569	Upregulated in HCC	*In vitro* and *in vivo*	Huh7, HepG2	Proliferation (+), migration (+), invasion (+)	Circ_001569/miR-411-5p/miR-432-5p	(54)
CircRNA101368	Upregulated in HCC	*In vitro*	HepG2, HCCLM3	Migration (+)	CircRNA 101368/miR-200a/HMGB1	(55)
Hsa_Circ_101280	Upregulated in HCC	*In vitro* and *in vivo*	HepG2, SNU-398	Proliferation (+), apoptosis (−)	Hsa_Circ_101280/miR-375/JAK2	(56)
Has_circ_0016788	Upregulated in HCC	*In vitro* and *in vivo*	Hep3B, Huh7	Proliferation (+), invasion (+), apoptosis (−)	Hsa_Circ_0016788/miR-486/CDK4	(57)
Hsa_circ_0103809	Upregulated in HCC	*In vitro*	HepG2, Huh7	Proliferation (+), migration (+), apoptosis (−)	Hsa_Circ_0103809/miR-490-5p/SOX2	(58)
CircNFATC3	Downregulated in HCC	*In vitro* and *in vivo*	HepG2, Huh7	Proliferation (−), invasion (−), migration (−), apoptosis (+)	CircNFATC3/miR-5481/NFATC3	(59)
CircMTO1	Downregulated in HCC	*In vitro*	HepG2, Hep3B	Proliferation (−), migration (−), apoptosis (+)	CircMTO1/miR-9-5p/NOX1	(60)
Circ-0051443	Downregulated in HCC	*In vitro* and *in vivo*	Huh7, Hep3B	Proliferation (−), apoptosis (+)	Circ-0051443/miR-331-3p/BAK1	(61)
CircARSP91	Downregulated in HCC	*In vitro* and *in vivo*	HepG2, MHCC97H, SK-Hep1	Proliferation (−), invasion (−)	AR/ADAR1/CircARSP91	(62)
cSMARCA5	Downregulated in HCC	*In vitro* and *in vivo*	SMMC7721, HCCLM3	Proliferation (−), migration (−)	cSMARC5/miR-17-3p/miR-181b-5p/TlMP3	(63)
CircADAMTS13	Downregulated in HCC	*In vitro* and *in vivo*	PLC-REF-5, HepG2	Proliferation (−), apoptosis (+)	CircADAMTS13/miR-484	(64)
CircADAMITS14	Downregulated in HCC	*In vitro* and *in vivo*	SK-HEP-1	Proliferation (−), invasion (−), apoptosis (+)	CircADAMTS1/miR-572/RCAN1	(65)
CircC3P1	Downregulated in HCC	*In vitro*	Hep3B, MCC97L	Proliferation (−), invasion (−), migration (−)	CircC3P1/miR-4641/PCK1	(66)
CircHIAT1	Downregulated in HCC	*In vitro* and *in vivo*	Hep3B, HCCLM3	Proliferation (−), apoptosis (+)	CircHIAT1/miR3171/PTEN	(67)
CircLARP4	Downregulated in HCC	*In vitro* and *in vivo*	MHCC97L, HCCLM3	Proliferation (−)	CircLARP4 miR-761/RUNX3/p53/p21	(68)
CircSETD3	Downregulated in HCC	*In vitro* and *in vivo*	Huh7, Hep3B	Proliferation (−)	CircSETD3/miR-421/MAPK14	(69)
CircTRIM33-12	Downregulated in HCC	*In vitro* and *in vivo*	SMMC7721, HCCLM3	Proliferation (−), invasion (−), migration (−)	CircTRIM33-12/miR-191/TET1	(70)
Hsa_circ_103809	Downregulated in HCC	*In vitro*	Hep3B, QGY-7703	Proliferation (−), invasion (−), migration (−)	Hsa_Circ_103809/miR-620	(71)

Ref, reference; EMT, epithelial to mesenchymal transition; TAM, tumor associated macrophage; (+) means promoting effect; (−) means inhibiting effect.

### CircRNA Inhibits Tumor Progression in HCC

CircRNAs not only promote the progression of HCC but also delay HCC progression (**Table 1** and **Figure 3**). CircNFATC3 is downregulated in HCC and impedes HCC development (59). Wang et al. (60) reveal that circMTO1 is low-expressed in HCC, and overexpression of circMTO1 inhibits the proliferation and migration and induces the apoptosis of HCC cells. Circ-0051443, a circRNA that is transported from normal cells to HCC cells *via* exosomes, suppresses HCC progression by sponging miR-331-3p to induce the expression of BAK1 (61). CircARSP91, a circRNA that is suppressed by AR in an ADAR1-dependent manner, functions as a tumor suppressor in HCC (62). Yu et al. (63) indicate that cSMARCA5 promotes the expression of TlMP3 (a well-known tumor suppressor) by binding to miR-17-3p and miR-181b-5p, and then slower the growth of HCC. Qiu et al. (64) perform an extensive analysis of circRNA profiles in 10 HCC tumor tissues and paired nontumor tissues and discover that circADAMTS13 is remarkably downregulated in HCC tissues. Overexpression of circADAMTS13 in HCC cells inhibits cell proliferation and promotes cell apoptosis by sponging miR-484. Song et al. (65) reveal that circADAMITS14 induces HCC cell apoptosis and inhibits HCC cell proliferation and invasion by regulating miR-572/RCAN1. Zhong et al. (66) find that circC3P1 suppresses the growth and metastasis of HCC by sponging miR-4641 to enhance PCK1 expression. CircH1AT1 is demonstrated to inhibit tumor growth in HCC *in vivo* and *in vitro* by targeting the miR-3171/PTEN axis (67). CircLARP4 is found to suppress tumor growth in HCC by mediating the miR-76/RUNX3/p53/p21 signaling pathway (68). Xu et al. (69) demonstrate that circSETD3 restrain the proliferation of HCC cells by blocking HCC cells in the G1/S phase. Zhang et al. (70) show that the decreased expression of circTRIM33-12 promotes tumor proliferation, migration, invasion, and immune evasion by binding to miR-191 to upregulate TET1 expression. It is reported that hsa_circ_103809 is significantly downregulated in HCC tissues and cell lines and acts as a miRNA sponge of miR-620 to inhibit the proliferation and invasion of HCC cells (71).

So far, people’s cognition and understanding of the correlation between circRNA and HCC is just the tip of an iceberg, and many unknown fields need to be studied. For example, some studies have observed that circRNA can induce DNA methylation or demethylation of CpG island of gene promoters, leading to the activation or inactivation of key genes or signaling pathways, thereby affecting disease development (16, 76). A recent study reveals that circRNA can undergo N6-methyladenosine (m6A) modification, which promotes protein translation (77). However, the relationships between circRNA and DNA methylation and m6A modification have not been elucidated in HCC. Whether circRNA can be modified by m6A or affect the methylation of other genes in HCC deserves further study.

## Clinical Significance of circRNA in HCC

### Application of circRNA in the Diagnosis of HCC

Increasing studies reveal that circRNA shows good performance in differentiating HCC patients from normal controls, indicating that it is expected to be a biomarker for HCC diagnosis.

Wu et al. (78) reveal that circ_0009582, circ_0037120 and circ_0140117 are increased in HBV-related HCC samples comparing with either chronic hepatitis or healthy controls. Combination of the three circRNAs and AFP can be employed to predict the occurrence HCC with HBV infection. Cheng et al. (79) report that circ_0016788 is upregulated in HCC tissues and it shows an excellent performance in distinguishing HCC samples from normal controls (area under the curve (AUC): 0.913). Circ-0051443 is mainly packaged in exosomes in HCC (61). The receiver operating characteristic (ROC) curve shows that the exosomal circ-0051443 can distinguish HCC patients from normal controls (ACU: 0.8089). CircRNA_104075 is highly expressed in HCC and shows an excellent diagnostic value in HCC patients, with an AUC value of 0.973 (80). Guan et al. (57) demonstrate a high diagnostic value of hsa_circ_0016788 in HCC (AUC=0.851).

In addition to in HCC tissues, abnormally expressed circRNA can also be detected in peripheral blood of patients with HCC, which provides a more convenient idea for the development of the diagnostic value of circRNA. Sun et al. (81) report that hsa_circ_0004001, hsa_circ_0004123 and hsa_circ_0075792 are upregulated in the blood samples of HCC. ROC analysis shows that the AUC value of the combination of the three circRNAs is 0.89, indicating that the combination of the three circRNAs could be a potential diagnostic biomarker in HCC. Lei et al. (82) conducted an RNA-seq analysis to identify differently expressed circRNAs in peripheral blood mononuclear cells (PBMCs) of HCC patients and normal controls. They find that circ_0000798 can distinguish HCC patients from healthy controls, suggesting that it may be a promising biomarker that can be detected in blood for the diagnosis of HCC. The above evidences indicate that circRNAs are expected to be used clinically for HCC diagnosis.

### Application of circRNA in the Prognosis of HCC

CircRNA is highly tissue-specific and more stable than linear mRNA, making it a promising prognostic marker for HCC. Circ_0005394 is upregulated in HCC, and overexpression of circ_0005394 is linked with larger tumor size, more advanced TNM stages, and poorer survival outcome for HCC patients (35). An onco-circRNA, circMET, is identified to be an independent predictor of overall survival and postoperative recurrence in HCC patients (38). Circ_0000267 is reported to be closely related to the clinical severity and worse survival outcome of patients with HCC (83). Chen et al. (75) show that the upregulated has_circ_0128298 is an independent factor for the prognosis prediction in patients with HCC. An oncogenic circRNA, circSLC3A2, is overexpression in HCC tissues and is associated with poor survival outcome in HCC patients (84). CircSETD3 is downregulated in HCC tissues, low expression of circSETD3 indicates shorter survival time in patients with HCC (69). The previous report shows that tumor patients with high tumor-infiltrating lymphocytes (TILs) have favorable survival outcome. Weng et al. (85) perform a microarray analysis by using plasma of HCC patients with high- and low-TILs and find that hsa_circ_0064428 is downregulated in patients with high TILs and is negatively related to overall survival, indicating that hsa_circ_0064428 is a promising immune-associated prognostic marker for patients with HCC.

A biomarker that can be detected from peripheral blood and other body fluids by non-invasive methods has better clinical application value. A study conducted by Gong et al. (46) indicates that circ-ZEB1.33 is upregulated in HCC tissues and predicts unfavorable prognosis in HCC patients. In addition, the authors demonstrate that circ-ZEB1.33 can also be detected in patients’ serum and that its expression in the serum of HCC patients is remarkably higher than that in healthy control. More importantly, the high expression of circ-ZEB1.33 in the serum of HCC patients indicates a shorter survival time. These findings suggest that circ-ZEB1.33 expression can be detected clinically by non-invasive methods to assess the prognosis of HCC patients. CircRNA_101237 is identified to be upregulated in the serum of HCC patients as compared with that in normal controls, and high expression of serum circRNA_101237 predicts unfavorable clinical outcome, indicating that serum circRNA_101237 can be a promising biomarker for prognosis prediction in HCC patients (86).

### Application of circRNA in the Treatment of HCC

Although treatment methods have been developed, the therapeutic effect of HCC is yet not optimistic. In the past few decades, the exploration of circRNA in HCC provides a breakthrough for future HCC treatment.

On the one hand, circRNA regulates certain signaling pathways, such as mTOR signaling pathway (41), β-catenin signaling pathway (44), NOTCH2 signaling pathway (44), Wnt signaling pathway (48), and p53/p21 signaling pathway (68), which play critical roles in the progression of HCC. Changing circRNA expression leads to the activation or inactivation of these signaling pathways, thereby affecting the progression of HCC. On the other hand, circRNA activates or inactivates some oncogenes and tumor suppressor genes, affecting the evolvement of HCC. For example, circ-BIRC6 enhances the expression of Bcl2, a well-known oncogene, to promote HCC development (43). All these evidences indicate that circRNA plays a key role in the development of HCC. Increasing or inhibiting the expression of circRNA in HCC patients may reverse the progress of HCC. However, the technique of specifically altering the expression of a specified circRNA in human body still needs further study. Increasing studies show that nanomaterials are promising drug carriers for cancer therapy. Nano-based delivery of circRNA overexpression or RNA interference molecules into tumor tissues of HCC patients is expected to be used for molecular targeted therapy of HCC patients.

### Relationship Between circRNA and Chemotherapy Resistance in HCC

It is well known that drug resistance occurs in the chemotherapy of most cancers at a later stage, leading to unsatisfactory therapeutic effects. Recent studies have focused on the involvement of dysregulated circRNA in HCC drug resistance (**Figure 4**). Huang et al. (87) show that circFoxo3 is overexpressed in Adriamycin-resistant HCC tissues and cell lines and circFoxo3 may induce Adriamycin resistance *via* the miR-199a-5p/ABCC1 axis. CircUHRF1 is secreted by HCC cells and transports to plasma by exosomes, and a high level of exosomal circUHRF1 contributes to natural killer cell exhaustion and drives resistance to anti-PD1 immunotherapy in HCC (88). Zhou et al. (86) reveal that the expression of circRNA_101237 in the serum of cisplatin-resistant HCC patients and cisplatin-resistant Huh7 cells is elevated, indicating that circRNA_101237 contributes to cisplatin-resistant in patients with HCC. A study conducted by Chen et al. (89) shows that circ_0003418 is downregulated in HCC. Knockdown of circ_0003418 enhanced cisplatin resistance of HCC cells. Interestingly, the authors also find that silencing circ_0003418 results in the activation of the Wnt/β-catenin signaling cascade, and the promotion of circ_0003418 on the sensitivity of HCC cells to cisplatin is reversed after inhibiting Wnt/β-catenin pathway.

The above evidences show that circRNA is expected to be a prospective biomarker for the diagnosis and prognosis assessment of cancer patients. However, there are still several problems to be settled in the clinical application of circRNA as a biomarker. For example, the non-invasive examination has more excellent clinical application value, while most previous studies focus on the expression of circRNA in HCC tissues and only a few studies have focused on the expression of circRNA in peripheral blood, urine, saliva and other body fluids. In addition, circRNA has been demonstrated to be related to the chemotherapy resistance of HCC patients, so how to improve the sensitivity of traditional chemotherapeutics and immunotherapy by targeting circRNA clinically is worth attention. Therefore, we need to do more in-depth studies to determine the relationship between circRNA and HCC, exploring more accurate and straightforward detection methods, selecting more stable and suitable circRNA as a diagnostic and prognostic biomarker or treatment target in patients with HCC.

## Conclusion and Perspective

Growing studies have shown that circRNA is differently expressed in various human malignant tumors. Differentially expressed circRNA is involved in tumor development by functioning as miRNA sponges, interacting with RBPs, translating proteins, competing with linear mRNA splicing of pre-mRNA and regulating parental genes. In this study, we comprehensively tracked the latest progress of circRNA in the pathogenesis of HCC. The circRNA-related studies have brought us many surprising findings suggesting that circRNA participates in the pathobiology of HCC. However, there are some open questions. Firstly, thousands of circRNAs in HCC have been detected and identified by RNA-seq, but only a small amount of functional circRNA has been characterized in HCC. It is necessary to analyze the function of more circRNAs and to understand the cell location, transportation, and degradation of circRNAs, which will enrich our knowledge of the complex regulatory networks involved in hepatocarcinogenesis. Secondly, circRNA has been reported to be a promising diagnostic and prognostic marker of HCC, but most of the previous studies have focused on circRNAs those expressed in HCC tissues. In fact, circRNAs those secreted in peripheral blood, exosomes, urine, and saliva are easier to be detected clinically. Therefore, more study is needed to mine circRNAs exist in body fluids to develop circRNA detection kits for HCC screening and prognosis monitoring. In conclusion, more studies are needed to disclose the relationship between circRNA and HCC to promote the clinical application of circRNA in HCC diagnosis, prognosis prediction, and therapy.

## Author Contributions

Study concept and design: DX and GC. Analysis and interpretation of data: DX, RH, and YD. Drafting of the manuscript: DX and HW. Critical revision of the manuscript for important intellectual content: DX, RH, and GC. Obtained funding: ZF and GC. Study supervision: HW and GC. All authors contributed to the article and approved the submitted version.

## Funding

This study is supported by the Natural Science Foundation of Guangxi, China (2018GXNSFAA294025) and the Guangxi Degree and Postgraduate Education Reform and Development Research Projects, China (JGY2019050).

## Conflict of Interest

The authors declare that the research was conducted in the absence of any commercial or financial relationships that could be construed as a potential conflict of interest.

## Abbreviations

AUC, area under the curve; circRNA, cicular RNA; ciRNA, circular intronic RNA; ecircRNA, exonic circRNA; EIciRNA, exonic-intronic circRNA; EMT, epithelial-to-mesenchymal transition; HCC, hepatocellular carcinoma; HIF1AN, hypoxia-inducible factor asparagine hydroxylase; ICMs, ntron complementary sequences; PBMCs, peripheral blood mononuclear cells; pre-mRNA, precursor messenger RNA; PI3K, phosphoinositide 3-kinase; QKI, quaking; RBP, RNA-binding protein; RCMs, reverse complementary matches; ROC, receiver operating characteristic; TAM, tumor-associated-macrophage; TILs, tumor-infiltrating lymphocytes.
